# X‐ray Stain Localization with Near‐Field Ptychographic Computed Tomography

**DOI:** 10.1002/advs.202201723

**Published:** 2022-06-24

**Authors:** Kirsten Taphorn, Madleen Busse, Johannes Brantl, Benedikt Günther, Ana Diaz, Mirko Holler, Martin Dierolf, Doris Mayr, Franz Pfeiffer, Julia Herzen

**Affiliations:** ^1^ Chair of Biomedical Physics Department of Physics School of Natural Sciences Technical University of Munich 85748 Garching Germany; ^2^ Munich Institute of Biomedical Engineering (MIBE) Technical University of Munich 85748 Garching Germany; ^3^ Paul Scherrer Institute Villigen 5232 Switzerland; ^4^ Institute of Pathology Ludwig‐Maximilians‐University 80337 Munich Germany; ^5^ Department of Diagnostic and Interventional Radiology School of Medicine & Klinikum rechts der Isar Technical University of Munich 81675 München Germany; ^6^ Institute for Advanced Study Technical University of Munich 85748 Garching Germany

**Keywords:** contrast agents, ptychographic computed tomography, quantitative X‐ray imaging

## Abstract

Although X‐ray contrast agents offer specific characteristics in terms of targeting and attenuation, their accumulation in the tissue on a cellular level is usually not known and difficult to access, as it requires high resolution and sensitivity. Here, quantitative near‐field ptychographic X‐ray computed tomography is demonstrated to assess the location of X‐ray stains at a resolution sufficient to identify intracellular structures by means of a basis material decomposition. On the example of two different X‐ray stains, the nonspecific iodine potassium iodide, and eosin Y, which mostly interacts with proteins and peptides in the cell cytoplasm, the distribution of the stains within the cells in murine kidney samples is assessed and compared to unstained samples with similar structural features. Quantitative nanoscopic stain concentrations are in good agreement with dual‐energy micro computed tomography measurements, the state‐of‐the‐art modality for material‐selective imaging. The presented approach can be applied to a variety of X‐ray stains advancing the development of X‐ray contrast agents.

## Graphical abstract

A strategy for the study of X‐ray contrast agents in individual cells with ptychographic X‐ray computed tomography is proposed. In addition to the stain concentration, which is validated by a state‐of‐the‐art quantitative imaging modality, the biological structures to which the contrast media binds to can be assessed. By that, the development of X‐ray stains and corresponding staining protocols is advanced.

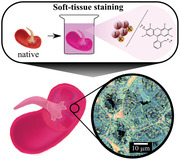

## 1 Introduction

X‐ray micro computed tomography (microCT) has become a valuable tool in many fields of application, providing 3D information of the sample's internal structure noninvasively. Especially for soft‐tissue visualization, microCT has contributed to many scientific findings.^[^ ^1^ ^]^ For instance, the visualization of small‐animal disease models has helped to understand disease progression.^[^ ^2^, ^3^ ^]^ Furthermore, besides developmental biology,^[^ ^4^, ^5^, ^6^ ^]^ microCT is widely used in drug development,^[^ ^7^ ^]^ imaging of insects in entomology,^[^ ^8^ ^]^ and virtual histology.^[^ ^9^, ^10^, ^11^, ^12^, ^13^ ^]^ The resolution in the micrometer range is usually sufficient to identify structural features such as the vascular system and bone structures, or to examine the interior of whole specimens (i.e., chicken embryos).^[^ ^6^, ^14^ ^]^

Early applications of microCT mainly focused on structures that inherently provide a high contrast.^[^ ^1^ ^]^ In case of soft‐tissue samples, however, the contrast in microCT is typically low due to the light elements present (hydrogen, carbon, oxygen, etc.). To overcome this limitation, heavy‐element X‐ray stains are used to increase the attenuation of the soft‐tissue samples and thus the contrast.^[^ ^15^ ^]^ Although this basic principle is simple, X‐ray contrast agents and corresponding staining protocols are constantly evolving.

In general, different types of interactions take place between a contrast agent and a biological target. Next to covalent bonding, several noncovalent interactions can occur such as ionic, halogen bonding, or the hydrophobic effect.^[^ ^16^, ^17^, ^18^ ^]^ In contrast to nonspecific contrast agents, several X‐ray stains have a chemical structure that binds to certain biological structures, such as proteins in the cell cytoplasm^[^ ^12^ ^]^ or the DNA in the cell nucleus.^[^ ^19^ ^]^

Although X‐ray contrast agents can be chemically structured to show a specific performance (e.g., specific targeting and/or absorption characteristics^[^ ^4^, ^5^, ^12^, ^19^, ^20^, ^21^, ^22^ ^]^), little is usually known about the interaction of a stain with the tissue, as well as its accumulation, and thus, concentration within the sample on a cellular level. Quantifying these parameters is difficult, since it requires high resolution and sufficient sensitivity to small amounts of contrast agent.

In this work, we demonstrate quantitative 3D near‐field ptychographic X‐ray CT (PXCT) as a technique for the assessment of the distribution and location of X‐ray contrast agents on a cellular level in murine kidney samples. For that, two different X‐ray stains are used. The first X‐ray stain is the inorganic iodine potassium iodide (IKI) (Scheme S1, Supporting Information). The triiodide and iodide anion bind to cationic structures in the tissue via ionic interactions. Iodine is the most used heavy element for X‐ray staining^[^ ^5^ ^]^ and suitable for a broad range of microCT applications.^[^ ^15^ ^]^ Advantageously, in addition to the ease of access and synthesis, IKI can also be used for larger samples (e.g., whole organisms^[^ ^14^ ^]^) by adjusting the reaction times during the staining procedure. The second stain investigated in this work is eosin Y (eosin), a novel X‐ray contrast agent, which originates from histopathology and is employed as a stain for light microscopy. The negatively charged eosin molecule contains four bromine atoms (Scheme S2, Supporting Information) and interacts noncovalently with the amino acid side chains of proteins and peptides. Eosin is known as a stain for the cell cytoplasm and is thus highly interesting for 3D virtual histology.^[^ ^12^ ^]^

The concentration of the two X‐ray stains in the bulk soft‐tissue samples was derived from dual‐energy microCT (microDECT) measurements, the state‐of‐the‐art imaging modality for the retrieval of material‐selective information by means of a basis material decomposition. Mathematically, this is performed by a change of basis vectors (cf., Experimental Section for details). In combination with an image‐based material decomposition, microDECT provides the stain concentration on a microscopic level.^[^ ^23^, ^24^ ^]^

PXCT combines raster scanning X‐ray microscopy, coherent diffractive imaging, and CT. By performing 2D coherent diffractive imaging for multiple projections, the full transmission function is acquired, and the real and imaginary parts of the complex‐valued refractive index can be retrieved. Therefore, the 3D attenuation coefficient and electron density distribution are obtained simultaneously. Especially for the visualization of unstained soft tissue the electron density usually provides a much higher contrast compared to the attenuation channel. The attenuation and electron density are two independent measures enabling a quantitative material decomposition, comparable to the microDECT standard, but, in our case, with a more than an order of magnitude higher resolution than that offered by conventional laboratory‐based microCT.^[^ ^25^, ^26^, ^27^, ^28^ ^]^

## 2 Results and Discussion

Two pairs for both X‐ray stains, each consisting of a stained and an unstained sample, were prepared from mouse kidneys. Structural similarity between the samples of each pair was ensured by the sample preparation procedure (see Experimental Section for details and Figure S1, Supporting Information).

All pairs were investigated with microDECT and subsequently with PXCT to localize the X‐ray stains on a cellular level. For presentation purposes, we focus on the results of one pair per stain (cf., Table S2, Supporting Information: IKI: CWI3; eosin: CWE2), however, the results presented apply to the second pairs consistently and their quantitative stain concentrations are given in brackets for comparison.

### 2.1 Microscopic X‐Ray Stain Quantification

Dual‐energy X‐ray imaging provides material‐selective information by exploiting the sample's attenuation at different X‐ray energies (or for different polychromatic X‐ray spectra), and subsequently enables a decomposition of the sample into desired basis materials based on prior knowledge of their energy‐dependent attenuation (see Experimental Section for details). For both X‐ray stains investigated in this work, one basis material was the heavy element present in the molecules (IKI: iodine; eosin: bromine). The major reason for the choice of this basis material was the reliability of the theoretical values for the energy‐dependent attenuation of the pure elements.

The samples had to be cryo‐ and vacuum‐compatible, which is why the sample preparation included critical‐point drying (CPD) of the soft tissue (cf., Experimental Section and Figure S1, Supporting Information). Although wet samples are generally not problematic for microCT, these measurements were also performed after the CPD procedure for comparability. The unknown attenuation coefficient of the basis material for soft tissue after CPD were retrieved from a measurement of an unstained sample (see Experimental Section for details).

**Figure 1:**
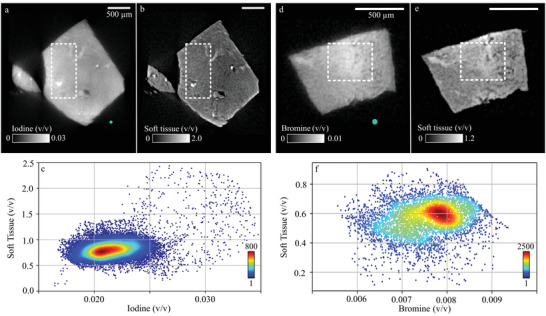
Dual‐energy micro computed tomography (microDECT) material decomposition of two samples, a–c) one stained with iodine potassium iodide (IKI), d–f) one with eosin Y (eosin). a,b) Iodine and critical point dried (CPD) soft‐tissue image of the sample stained with IKI, respectively. c) The scatterplot (sample size *N* = 1 × 264 × 84 voxels) shows the pixel‐wise iodine to CPD soft‐tissue volume fraction from a volume‐of‐interest (VOI) (dotted rectangle in (a) and (b)). d,e) Bromine image and CPD soft‐tissue image of the eosin‐stained sample, respectively. f) The scatterplot (*N* = 1 × 60 × 90 voxels) shows the pixel‐wise bromine to CPD soft‐tissue volume fraction (VOI depicted as dotted rectangle in (d) and (e)). The turquoise spots in (a) and (d) illustrate the size of the pillar imaged with ptychographic X‐ray CT (PXCT). All scalebars are 500 μm.

The material decomposition into iodine (**Figure** **1**a) and CPD soft tissue (Figure 1b) of the IKI‐stained sample results in a mean microscopic iodine volume fraction in a volume‐of‐interest (VOI) (indicated as dotted rectangle in Figure 1a,b; height one voxel) of (2.17 ± 0.04)% (v/v), for a soft‐tissue fraction of (83.4 ± 4.7)% (v/v) (Figure 1c) (pair 2: I: (1.97 ± 0.03)% (v/v); CPD soft tissue: (85.5 ± 4.5)% (v/v)).

The sample stained with eosin was decomposed into bromine (Figure 1d) and CPD soft tissue (Figure 1e). A mean bromine concentration of (0.75 ± 0.02)% (v/v) was found for a mean CPD soft‐tissue fraction of (57.0 ± 4.0)% (v/v) (Figure 1f) (pair 2: Br: (0.66 ± 0.02)% (v/v); CPD soft tissue: (50.83 ± 4.0)% (v/v)). The lower CPD soft‐tissue volume fractions indicate that the density of the samples stained with eosin is increased compared to the unstained reference. The applied image‐based material decomposition is a basis vector transformation which is mathematically performed by solving a linear set of equations with two unknowns (see Experimental Section for details) and intrinsically has a unique solution. The resulting material volume fractions give the amount of the respective material contained in the voxel, which restore the measured total attenuation coefficient in the low and high energy measurement. Therefore, volume conservation does not necessarily have to be fulfilled due to variations in the density of the soft tissue.

The reconstructed voxel size of 5.0 μm for both samples was sufficient to identify the tubule structure within the murine kidney samples (cf., Figure 1) and sufficient contrast was observed in both specimens. The contrast‐to‐noise ratio (CNR) increased by a factor of 3.08 (pair 2: 3.50) in the sample stained with IKI, and by a factor of 2.02 (pair 2: 1.82) in the sample stained with eosin, compared to the unstained sample (cf., CNR values provided in Table S2, Supporting Information).

The samples were evenly stained with the respective contrast agent. There is no gradient visible in the stain concentration through the 3D basis material volumina (cf., Figure S2, Supporting Information, which provides the histograms of the volume fractions of the respective contrast medium and soft tissue for every slice through the 3D basis material images). Thus, uneven staining can be excluded. Furthermore, the standard deviation in the whole sample volume of the iodine volume fraction was σ_I_ = 0.28% (v/v) (pair 2: 0.15% (v/v)) and σ_Br_ = 0.14% (v/v) (Pair 2: 0.11% (v/v)) of the bromine fraction. Both are small compared to the observed mean volume fraction of the respective staining element. It must be noted, that σ_Br_ and σ_I_ contain, besides the standard deviation in the signal itself, the difference in stain concentration in different sample structures.

The mean volume fraction of bromine was found to be lower compared to the one of iodine in the respective VOIs. The physical sizes of eosin and of the molecules of the IKI stain are not exactly known. However, in general the ionic radius of organic ions is larger compared to inorganic ions.^[^ ^29^ ^]^ Furthermore, the eosin molecule consists of far more atoms compared to the IKI molecules. The weight fraction of bromine in eosin is only 49.5% (w/w), whereas the one for iodine reaches 90.7% (w/w) in the case of KI_3_ and even 100% (w/w) in the I_2_ anion. Together with the presumably larger size of the eosin molecule, this makes it plausible that bromine does not accumulate in such a high concentration as iodine.

In summary, microDECT measurements demonstrated that both X‐ray stains provide a high contrast enhancement, and the samples were shown to be evenly stained. Because the same basis materials are applied in the following to determine the nanoscopic stain concentrations in PXCT, the microscopic stain concentrations are used as comparative values, since the mean volume fractions of both heavy elements are independent of the resolution, given uneven staining.

### 2.2 Quantitative Ptychographic X‐Ray Computed Tomography

PXCT measurements at the cSAXS beamline at the Paul Scherrer Institute are limited to a small volume of interest, which was prepared from the respective bulk sample by cutting the sample to the form of a pillar with diameters between 60 and 80 μm (size of turquoise circles in comparison to the bulk sample in Figure 1a,d; pillar diameters for each sample are given in Table S2, Supporting Information).^[^ ^30^ ^]^ No additional sample preparation was applied.

**Figure 2:**
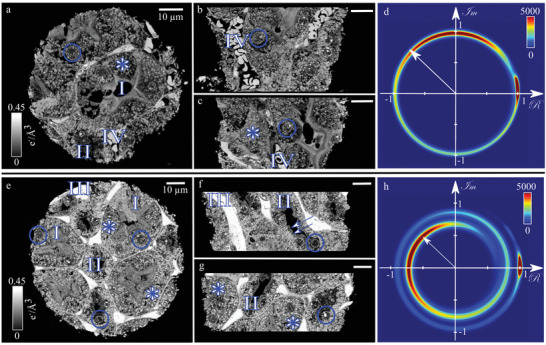
Comparison of unstained (top) and IKI‐stained murine kidney piece (bottom). a–c) Orthogonal slices through the 3D reconstructed electron density. The colorbar in (a) applies to (b) and (c). d) 2D histogram (*N* = 325.4 × 10^6^) of the measured real and imaginary part of the transmission function (projection data) of the unstained sample. The vector from the origin to a specific point on the circle corresponds to the total transmission in the pixel, which depends among others on the sample thickness. The transmission is slightly below one. e–g) Murine kidney sample stained with IKI. The contrast is higher compared to the unstained sample (a–c). The colorbar in (e) applies to (f) and (g). h) The histogram (*N* = 350.1 × 10^6^) of the stained sample shows that the transmission is significantly decreased due to the X‐ray stain (white vector). d,h) The peaks at (1+0*i*) correspond to the background (vacuum). All scalebars are 10 μm. Exemplified annotation: I: proximal tubules; II: distal tubules; III: blood capillaries; IV: erythrocytes; blue circles: cell nuclei; *: lysosomes; arrows in (f): basal striations.

Both samples of the IKI pair consist of two different types of tubules in the kidney. Standard histological images of a murine kidney are provided in Figure S3, Supporting Information, (e) and (f) with exemplified annotated structures, for comparison. Proximal tubules (**Figure** **2**, I) have a lumenal brush border of microvilli, microscopic cellular membrane protrusions that lead to an increase of the luminal surface area for better reabsorption and diffusion. The cell nuclei (Figure 2, circle) appear irregularly, which is typical for proximal epithelia. The distal tubules (Figure 2, II) are smaller compared to proximal tubules and clearly delimited to the lumenal side of the membrane. Because distal tubules are composed of smaller cells, a higher number of cell nuclei is visible.

Both types of tubules show their typical basal striations (Figure 2, arrows), membrane folds which contain mitochondria. The tubules are surrounded by connective tissue and blood capillaries (Figure 2, III) that contain single erythrocytes (Figure 2, IV). Lysosomes (Figure 2, *), which are responsible for intracellular degradation of macromolecules, are also evident. The presence of distal and proximal tubules suggests the sample location to be in the outer medulla of the kidney. Since the sample is very small, the probability of containing a glomerulus is small as well. Therefore, the sample may also originate from the renal cortex (cf., Figure S3, Supporting Information, where a part of a glomerulus is visible in the unstained sample of the second IKI pair). The comparison of the unstained sample (Figure 2 top) and the sample stained with IKI (Figure 2 bottom) with respect to their electron density distributions (Figure 2a–c,e–g, respectively) shows a general increase in contrast in the stained sample. The increase in the mean electron density was Δρ_e_ = 0.063*e* ^−^Å^−3^ (Pair 2: Δρ_e_ = 0.033*e* ^−^Å^−3^; standard deviation of the background peak: $\sigma_{\rho_{e}}=$ 0.004 *e* ^−^Å^−3^). Especially the lysosomes (Figure 2, *) and the capillaries (Figure 2, III) provide a higher signal compared to the unstained counterpart.

The influence of IKI on the absorption of the sample is directly visible in the complex‐valued transmission. In the complex‐plane histogram of the measured transmission function from all projections (cf., Equation (1)), the vector length from the origin to a specific data point gives the transmission. The unstained sample provides a circularly shaped histogram with a transmission slightly below 1 (Figure 2d, white vector), which corresponds to minor absorption. The transmission of the sample stained with IKI is strongly reduced by the contrast agent (Figure 2h, white vector). The histogram in Figure 2h is a spiral, along which the projected thickness of the sample increases (due to the cylindrical shape of the sample). The angular component in the histogram represents the phase shift. A single rotation around the origin corresponds to a 2π phase shift (cf., Figure 2d). Multiple turns forming the spiral shape indicate that the phase projections are heavily wrapped (cf., Figure 2h). The peak at (1+0*i*) in Figure 2d,h corresponds to the vacuum background, which shows neither absorption nor phase shift.

**Figure 3:**
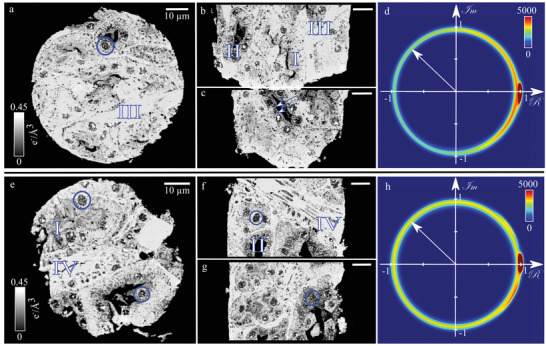
Comparison of two murine kidney samples: unstained (top) and stained with eosin (bottom). a–c) Orthogonal slices through the 3D reconstructed electron density of the unstained sample. Colorbar in (a) applies also to (b) and (c). d) 2D histogram (*N* = 378.5 × 10^6^) of the measured real and imaginary part of the transmission function of the unstained sample, generated from the projection data. The transmission is ≈1. e–g) Orthogonal slices of a murine kidney piece stained with eosin. Compared to the unstained sample, a small increase in electron density is present. Colorbar in (e) applies to (f) and (g). h) The histogram (*N* = 337.9 × 10^6^) of the stained sample shows that the overall transmission throughout the sample is not changed significantly by the X‐ray stain. d,h) The peak at (1+0*i*) corresponds to the background (vacuum). All scalebars are 10 μm. Exemplified annotation: I: proximal tubules; II: distal tubules; III: blood capillaries; IV: intermediate tubules; blue circles: cell nuclei; arrows in (c): basal striations.

The eosin pair of stained and unstained sample again shows the tubule structure inside the kidney. In addition to proximal (**Figure** **3**, I) and distal tubules (Figure 3, II), blood capillaries (Figure 3, III) are present. Intracellular features such as the cell nuclei (exemplary: Figure 3, circles) are visible. The stained sample also depicts an intermediate tubule (Figure 3, IV). Intermediate tubules consist of single‐layer squamous epithelium and the cell nuclei bulge inward. Their main function is to concentrate urine by water reabsorption. The presence of an intermediate tubule suggests that the samples originate from the renal medulla.

In both samples of the eosin pair, the contrast is not high. The shift in electron density of with Δρ_e_ = 0.01*e* ^−^Å^−3^ (Pair 2: Δρ_e_ = 0.01*e* ^−^Å^−3^; calculated from the mean electron density; standard deviation of the background peak: $\sigma_{\rho_{e}}=$ 0.004 *e* ^−^Å^−3^) between the sample stained with eosin and its unstained counterpart is small. The complex‐plane histogram of the projection data of the unstained sample (Figure 3d) shows almost no absorption and is in general very similar to the unstained sample from the IKI pair (cf., Figure 2d), as expected. The histogram of the stained sample (Figure 3h) shows that the contrast medium does not cause a significant decrease in transmission (cf., Figure 3, arrows).

In summary, the eosin‐stained sample is generally very similar to the unstained one, while the sample stained with IKI clearly differs from its unstained counterpart in both electron density and attenuation. The second sample pair of each X‐ray stain behaved like the presented ones (cf., Figure S4, Supporting Information).

The electron density of the eosin anion (estimated for the chemical formula of eosin Y according to ref. [31] $C_{20}H_{6}{\text{Br}}_{4}O_{5}^{2-}$) is 0.442 *e* ^−^Å^−3^. This is only slightly higher than the maximum electron density in the eosin pair's unstained sample of 0.399 *e* ^−^Å^−3^ (pair 2: 0.399 *e* ^−^Å^−3^). In contrast, the electron density of IKI (estimated to be 0.778 *e* ^−^Å^−3^^[^ ^31^ ^]^) is significantly higher than the electron density in the unstained samples. Thus, an increase in contrast in the samples stained with IKI is achieved. A reason for the lower absorption of the eosin‐stained sample compared to the sample stained with IKI (cf., vectors in Figures 2h and 3h) may be the smaller uptake of bromine compared to iodine, as suggested by the microDECT measurements. Furthermore, the atomic number of bromine (*Z*_Br_=35) and its attenuation is much lower than that of iodine (*Z*_I_ =53) at an X‐ray energy of 6.2 keV, which is closely above the L‐III‐edge, L‐II‐edge, and L‐I‐edge of Iodine (4.56, 4.85, and 5.19 keV, respectively), but below the bromine K‐edge at 13.47 keV (cf., Figure S5, Supporting Information).^[^ ^31^ ^]^

Differences in electron density and especially in contrast are visible between the unstained samples of both stains (cf., Figures 2e–g and 3e–g), which are potentially caused by the sample preparation. First, the samples may originate from different regions of the kidney and thus can differ in their density and composition. Since the pillars were very small, an exact localization of the imaged regions within the murine kidney is difficult. However, it is very likely that the samples of the IKI pair originate from the renal cortex and the samples of the eosin pair from the outer medulla. Phase‐contrast measurements of an unstained mouse kidney stored in agarose already showed variations in electron density between the different kidney regions.^[^ ^32^ ^]^ Specifically, an increased electron density was found in the outer medulla compared to the renal cortex. Second, all pairs of one X‐ray stain passed through the sample preparation procedure together, but separately from the samples of the other contrast agent. Therefore, we cannot entirely exclude the possibility that, despite conscientious work, different conditions existed during dehydration or CPD for the different sample pairs, which may have affected the samples differently. The macroscopic brown coloration of the IKI samples and the pink coloration of the eosin samples in comparison to a light color of the unstained samples rule out the possibility of sample mix‐ups.

### 2.3 Nanoscopic X‐Ray Stain Localization

The reconstructed attenuation exhibited stronger noise than the electron density distribution, which would propagate from the original data into the basis material images. Thus, we applied a joint dictionary denoising algorithm on both the reconstructed electron density and attenuation data that benefits from the structural features being the same in both dataset (for details see Experimental Section and Figure S6, Supporting Information).^[^ ^33^ ^]^

The heavy elements in the stains, iodine, and bromine, again served as the basis materials for the contrast agents. The mean attenuation coefficient and the mean electron density of the unstained samples served as the reference values for the CPD soft‐tissue basis material of each sample pair's stained specimen. This procedure is necessary because attenuation and electron density may vary from sample pair to sample pair (cf., Figures 2a–c and 3a–c).

**Figure 4:**
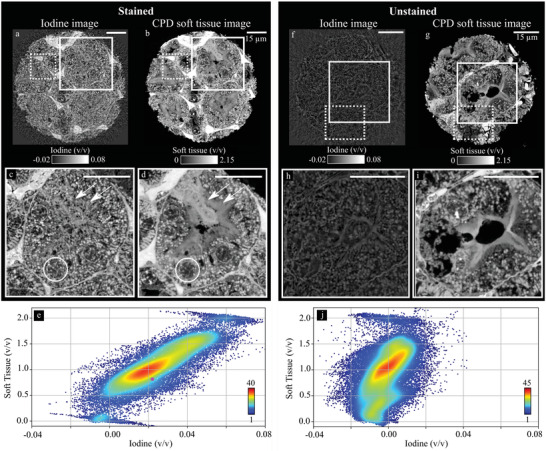
High‐resolution material decomposition of an IKI‐stained sample compared to the unstained counterpart. a,b) Murine kidney sample stained with IKI decomposed into iodine and CPD soft tissue, respectively. The zoom‐ins (c,d) solid rectangle in (a) and (b)) show the distribution of the iodine in a proximal tubule. Colorbars in (a) and (b) also apply to (c) and (d), respectively. The circle highlights a cell nucleus. The doubled arrow points toward the brush border of the tubules. e) Scatterplot (*N* = 1 × 175 × 155 voxels) of iodine versus CPD soft‐tissue fraction from a VOI (dotted rectangle in (a) and (b)). The pink dot marks the mean stain concentration retrieved from the bulk sample by the microDECT measurements (cf., Figure 1c). f,g) Decomposition of the unstained sample with zoom‐ins in (h) and (i), respectively. Colorbars in (f) and (g) apply to (h) and (i), respectively. j) The scatterplot (*N* = 1 × 220 × 180 voxels) shows a mean iodine fraction much smaller than of the stained sample, around 0. All scalebars are 15 μm.

The decomposition into iodine and CPD soft tissue of the sample stained with IKI (**Figure** **4**a,b) yields a much higher iodine concentration compared to the unstained counterpart (Figure 4f,g, zoom‐in of a proximal tubule for comparison shown in Figure 4h,i). The CPD soft‐tissue images of stained (Figure 4b) and unstained samples (Figure 4g) look qualitatively very similar. The cell nuclei in the stained sample (circle in Figure 4c) differ only slightly in their iodine concentration compared to the cell cytoplasm but are clearly differentiable in the soft‐tissue image (circle in Figure 4d). Furthermore, the brush borders of the tubules show a lower contrast in the iodine image than in the CPD soft‐tissue image, but the individual microvilli have interacted with iodine (double arrows in Figure 4c,d). Especially the lysosomes show an increased iodine volume fraction. Since lysosomes have an acidic pH value inside, the anionic *I*_2_ can potentially bind to many cationic structures. The extracellular iodine fraction is slightly higher than the intracellular one.

For the stained sample, the mean fractions of iodine and CPD soft tissue in the marked VOI are (2.5 ± 0.7)% (v/v) and (106.1 ± 2.4)% (v/v) (pair 2: (1.8 ± 0.4)% (v/v); (120.2 ± 2.2)% (v/v)) (Figure 4e). The results are in good agreement with the results from microDECT, where the iodine fraction was (2.17 ± 0.04)% (v/v) (pink dot in Figure 4e) (pair 2: (1.97 ± 0.03)% (v/v)).

The unstained sample has a mean iodine volume fraction of (−0.0 ± 0.4)% (v/v) at a mean CPD soft‐tissue fraction of (93.0 ± 1.7)% (v/v) (pair 2: (−0.2 ± 0.9)% (v/v); (97.6 ± 4.0)% (v/v)) (Figure 4j). Because variations in electron density and attenuation are present in the unstained sample, the iodine fraction slightly fluctuates around zero. The iodine image of the unstained sample (cf., Figure 4f,h) contains structural information of the areas that have a higher electron density and attenuation (cf., Figure 2a–c: e.g., erythrocytes, lysosomes, membranes, and structures in the cell nuclei). This comes from the assumption of a distinct electron density and attenuation coefficient for the CPD soft tissue.

The nanoscopic CPD soft‐tissue fractions of the samples are higher compared to the microscopic fraction (and more closely to 100%, depending to the VOI). The attenuation of the unstained soft‐tissue reference in microDECT was retrieved from a large region‐of‐interest, which can include different structures with varying attenuation and density. The unstained CPD soft‐tissue reference in PXCT, however, had very similar structures as in the stained sample and is thus expected to fit very well, especially in terms of the density. However, local density variations are also present on the nanoscopic scale and thus, volume conservation is not fulfilled.

In summary, the iodine in the IKI‐stained sample is evenly distributed inside the cells without conspicuous accumulation in specific areas. Structures in the cytoplasm (e.g., lysosomes) as well as the cell nuclei and the extracellular capillaries show similar iodine concentrations, which, on average, quantitatively agree well with the microscopic stain concentration derived from microDECT measurements.

**Figure 5:**
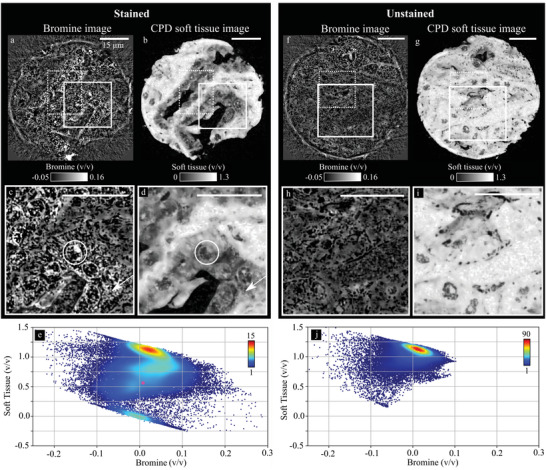
High‐resolution material decomposition of a murine kidney sample stained with eosin compared to the unstained counterpart. a,b) Murine kidney sample stained with eosin decomposed into bromine and CPD soft tissue. The zoom‐ins (c,d) solid rectangle in (a) and (b)) show the distribution of the bromine in a distal tubule. Colorbars in (a) and (b) also apply to (c) and (d), respectively. The single arrow highlights an increased bromine fraction in the cell cytoplasm. The circle highlights a cell nucleus, which also shows an increased bromine fraction. e) Scatterplot (*N* = 1 × 190 × 260 voxels) of the bromine versus CPD soft‐tissue fraction from a VOI (dotted rectangle in (a) and (b)). The pink dot marks the mean stain concentration retrieved from the bulk sample with the microDECT measurements (cf., Figure 1f). f,g) Decomposition of the unstained sample with zoom‐ins in (h) and (i), respectively. The size of the zoom regions is chosen such that they have the same size for the stained and the unstained sample. Colorbars in (f) and (g) apply to (h) and (i), respectively. All scalebars are 15 μm. j) The scatterplot (*N* = 1 × 200 × 200 voxels) of bromine to CPD soft‐tissue fraction in the unstained sample.

Although the difference in electron density and attenuation between the stained and unstained sample of the eosin pair were minor, differences in the bromine volume fraction between unstained (**Figure** **5**f) and eosin‐stained sample (Figure 5a) are visible. Figure 5 shows the same samples as in Figure 3 but the stained sample is depicted at a different horizontal position. While an increased bromine fraction can be found especially in tubular epithelia, but not in the intracellular region (Figure 5a,c). The bromine fraction is also increased in the cell nuclei. The unstained sample has no bromine content (Figure 5f,h). The decomposition of the unstained sample of the eosin leads to structures in the bromine image. They originate from increased electron densities and attenuation in the respective structures and are comparable to the structures present in the iodine image of the unstained sample of the IKI pair (cf., Figure 4f).

The mean bromine fraction in the VOI (dotted rectangle in Figure 5a,b) is (1.1 ± 2.2)% (v/v) (pair 2: (0.32 ± 1.8)% (v/v)) with a mean CPD soft‐tissue fraction of (79.1 ± 4.4)% (v/v) (Figure 5e) (pair 2: (96.5 ± 3.8)% (v/v)). In comparison, the mean bromine fraction in the unstained sample was (−0.3 ± 1.8)% (v/v) (pair 2: (−0.27 ± 2.2)% (v/v)) with a mean CPD soft‐tissue fraction of (103.5 ± 3.5)% (v/v) (Figure 5j) (pair 2: (97.3 ± 4.6)% (v/v). In comparison to the microDECT measurements (pink dot in Figure 5e), the mean bromine volume fraction for the stained samples of both pairs shows the same trend (Pair 1: (0.75 ± 0.02)% (v/v); pair 2: Br: (0.66 ± 0.02)% (v/v)). However, the mean bromine fraction from both microDECT and PXCT are smaller than the standard deviation in the basis material images retrieved from PXCT.

Although the standard deviations in the original data of the eosin and IKI sample pairs were similar, they strongly vary for the different basis material decompositions. In general, the standard deviations in the basis material images depend on the choice of the basis materials and the standard deviations in the original data (for more detail see Experimental Section). Although noise in the original data can be reduced (e.g., by increasing the measurement time or the X‐ray flux), PXCT, as well as other phase‐imaging techniques, suffers in general from the trade‐off between phase‐sensitivity and resolution. Increasing the resolution (reconstructed voxel size for all samples 99 nm) will decrease the sensitivity. Furthermore, the higher the resolution, the less amount of contrast agent is present per voxel requiring even higher sensitivity.

Another limitation accompanying the high resolution is the small field‐of‐view. Although for the investigation of the stains presented in this work the small field‐of‐view was not critical, it can become a strong limitation when larger biological structures are of interest, while maintaining the resolution (e.g., imaging of a whole glumerulus with a diameter of ≈70 μm). This also causes difficulties for the sample preparation as the regions‐of‐interest in the bulk sample have to be determined and prepared accurately. In general, the sample preparation procedure in this work was sophisticated. This was on the one hand due to the preparation of additional unstained samples from the same kidney regions, which is not absolutely necessary for the application of PXCT for the location of other X‐ray stains, but rather served as reference structures in this work. On the other hand, the cryogenic sample environment requires either frozen hydrated or dehydrated and dried samples.

Although laboratory‐based dual‐energy CT can be performed at higher resolutions compared to the demonstrated microDECT measurements (even in the same order of magnitude as in the PXCT measurements), PXCT should be the preferred quantitative imaging technique for the visualization of individual cells. The demonstrated image quality and resolution, which can even be increased when transferred to far‐field PXCT, in combination with the quantitative accuracy are not achievable with laboratory systems so far. Since X‐ray tubes are employed in high‐resolution laboratory‐based dual‐energy systems, their spectra are polychromatic. Therefore, assumptions about the mean energy must be introduced which result in uncertainties for the image‐based material decomposition. Moreover, they are inaccurate, especially with the mean energy of the spectrum being close to a material's K‐edge (e.g., iodine with a K‐edge at 33.2 keV^[^ ^31^ ^]^). Furthermore, attenuation and electron density obtained from the PXCT measurement are perfectly registered spatially, because both contrast channels are retrieved from the same measurement. In contrast, two measurements are required for dual‐energy imaging. As a consequence, already small sample drifts corrupt the spatial registration of dual‐energy measurements at high resolution.

## 3 Conclusion

We have determined the concentration and location of two different X‐ray stains quantitatively on a cellular level at a resolution of around 100 nm using PXCT. Although both X‐ray stains provided high contrast enhancement, the differences in accumulation and location of the IKI and the eosin inside the cells were substantial. The material‐selective analysis of the samples was based on the comparison between stained and unstained samples with similar structural features.

The basis material images of the stained samples revealed the location of the X‐ray stain in the cells. The proposed approach for the localization of X‐ray stains may be limited in case of a very low amount of contrast per voxel due to the noise present in the absorption data and its enhancement induced by the basis vector transformation. However, generally the sensitivity and resolution of near‐field PXCT was sufficient to resolve the small amounts of contrast media per voxel in intracellular structures, for example, the cell nuclei and lysosomes. A transfer to higher resolutions in far‐field PXCT is in general possible.

PXCT can potentially be applied to a variety of X‐ray stains as well as soft‐tissue samples other than the kidney. In addition to a better understanding of contrast agents and their interaction with the tissue, the development, and optimization of novel X‐ray stains and corresponding staining protocols benefits from the knowledge about their exact location inside the tissue.

## 4 Experimental Section

### Animals

Mouse kidneys served as soft tissue samples. The variety of biological structures within this organ (e.g., the cortex with its glumeruli or the medulla with its tubular system) made it possible to investigate several aspects of the staining procedure, for instance regarding completeness and homogeneity. Animals were housed at the Klinikum rechts der Isar, Technical University of Munich, in accordance with the European Union guidelines 2010/63. Organ removal was approved by an internal animal protection committee of the Center for Preclinical Research of the Klinikum rechts der Isar, Munich, Germany (internal reference number 4005‐09). All procedures were in accordance with relevant guidelines and regulations. All laboratories were inspected for accordance with the Organization for Economic Co‐operation and Development (OECD) principles of good laboratory practice. In this work, only murine kidney tissue was further investigated.

### Sample Preparation

The murine kidneys were surgically removed and immediately placed in 50 mL Falcon centrifuge tubes (neoLab), which were filled with a fixative solution containing 10 mL of 4% (v/v) formaldehyde solution (FA; derived from a 37% acid‐free FA solution stabilized with 10% methanol from Carl Roth; further dilution with Dulbecco's phosphate‐buffered saline (DPBS; Thermo Fisher Scientific) without calcium and magnesium). The samples were refrigerated for 24–72 h. The soft‐tissue samples were always prepared as pairs of stained and unstained soft tissue (Figure S1, Supporting Information). In total, two soft‐tissue pairs per stain were prepared. To ensure similar morphologic structures among the sample pairs, for each pair a tissue piece of 2 mm × 1 mm × 1mm was resected from a mouse kidney with a scalpel (Aesculap). Each cuboid was cut in half and the opposite faces of the shared face of the obtained 1 mm × 1 mm × 1 mm cubes were labeled. One of the cubes was stained while the other cube was kept unstained in FA solution at room temperature for the entire staining time of the paired cube. Afterward they were subjected to CPD and subsequently mounted to OMNY pins with the labeled faces.^[^ ^30^ ^]^ The surface of every sample was trimmed by cylindrical turning such that a VOI in form of a pillar remained.^[^ ^34^ ^]^ The pillars' diameter ranged from 60 to 80 μm (Table S2, Supporting Information).

### Eosin Y Staining Protocol:

The staining with eosin was performed according to ref. [12]. The cubic soft‐tissue samples were transferred to a small petri dish (diameter: 35 mm; height: 10 mm, Greiner) holding 2 mL of FA solution. To encourage the accumulation of eosin in the cytoplasm, in a first step, the soft‐tissue sample was acidified with glacial acetic acid (Alfa Aesar) by adding 5% (v/v) of glacial acetic acid (Alfa Aesar) to the petri dish. The samples acidified for 24 h and were then washed with DPBS solution for 1 h. The cubes were placed in 2 mL staining solution of eosin Y disodium salt (30% (w/v) in distilled water; Sigma‐Aldrich, product number E4382, stain certified by the Biological Stain Commission) for 24 h. During the incubation time, the samples were kept on a horizontal shaking plate allowing for a smooth rocking of 30 rpm. After staining, the soft‐tissue samples were carefully removed from the sample container and excess of staining agent was softly patted with a cellulose tissue paper.

### IKI Staining Protocol:

The staining with iodine potassium iodide (Lugol's solution) was adapted from ref. [6]. The mouse kidney tissue cubes were transferred to small petri dishes (diameter: 35 mm; height: 10 mm, Greiner) each holding 2 mL of a freshly prepared 2% aqueous IKI solution (iodine and potassium iodide from Sigma Aldrich). The samples were incubated in the staining solution for 24 h and then carefully removed from the petri dish. Excess of staining agent was softly patted with a cellulose tissue paper and the soft‐tissue samples were washed in DPBS for 1 h.

### Critical Point Drying:

When performing imaging experiments aiming at high resolution, the stability and accuracy of the sample position are crucial. For correct positioning and prevention of sample movements, the ptychography setup at the Paul Scherrer Institute used position tracking based on laser interferometry and a sample environment under cryogenic conditions in vacuum.^[^ ^35^, ^36^ ^]^ To avoid radiation damage and the formation of ice crystals, the sample preparation procedure included a dehydration series as well as CPD (Figure S1, Supporting Information).

The stained and unstained soft‐tissue cubes of 1 mm edge length were first dehydrated and underwent CPD afterward as described in ref. [12]. Briefly before the first dehydration step, each cube was transferred to a new petri dish, where they remained for all subsequent steps. For the dehydration series, 2 mL of the following concentrations (%v/v) were used: 50, 60, 70, 80, 90, 96, and 100 ethanol balanced with distilled water. Each dehydration incubation was performed for 15 min. The dehydrated murine kidney cubes were then transferred to CPD (Bal‐TEC CPD 030 with CO_2_ as drying agent). The CPD soft‐tissue cubes were stored in a petri dish before they were mounted on OMNY pins.^[^ ^35^, ^36^ ^]^

### MicroDECT Measurements:

The microCT system was the SkyScan1275 (Bruker, USA). Low energy measurements were performed with a tube voltage of 25 kV, a current of 125 μA, and an exposure time of 212 ms. The high energy measurement used a tube voltage of 40 kV and a tube current of 70 μA. The exposure time was 171 ms. 1565 projections were acquired over 360 degree for a single tomogram. The reconstructed voxel size was 5 μm as provided by the build‐in data acquisition software of the Skyscan 1275 (Bruker, USA). Mean energies for the decomposition were estimated from the attenuation of a polyoxymethylene (POM) rod. Comparing the measured attenuations of (1.95 ± 0.03) and (1.13 ± 0.02) cm^−1^ for low and high energy measurement with the theoretical attenuation of POM^[^ ^31^ ^]^ resulted in a mean energy of 14.75 keV for the low energy measurement, and 18.40 keV for the high energy measurement.

### Ptychographic X‐Ray Computed Tomography–Data Acquisition:

Experiments were performed with the OMNY setup at the cSAXS (X12SA) beamline of the Swiss Light Source at the Paul Scherrer Institute, which was designed specifically for nano‐tomography of cryogenically cooled specimens with X‐ray ptychography.^[^ ^35^ ^]^ The X‐ray energy was monochromatized with a Si(111)‐crystal monochromator to 6.2 keV. A horizontal slit system upstream of the monochromator reduced the apparent horizontal source size such that the horizontal and vertical coherence length were larger than the Fresnel zone plate used to define the coherent illumination on the sample.

The Fresnel zone plate had a diameter of 220 μm and an outermost zone width of 60 nm. The sample was placed 9.9 mm downstream of the focus, where the beam had a diameter of about 32 μm. The coherent diffraction patterns were acquired with an Eiger 1.5M detector,^[^ ^38^, ^39^ ^]^ which was located about 7.2 m behind the sample. Before the acquisition of the tomographic data, an initial ptychographic scan was recorded. The reconstructed illumination was used as the initial guess for the probe during reconstructions. The number of projections for each sample measurement were chosen according to the horizontal extent of the respective specimen (Table S2, Supporting Information). The acquisition time for a single diffraction pattern was 100 ms. For each projection the sample was raster‐scanned along a Fermat‐spiral, which was cut to a rectangular field‐of‐view, with a step size of about 4 μm, to avoid artifacts caused by the symmetry of a conventional raster grid scan.^[^ ^40^ ^]^ The scan was performed for angles ranging from 0 to 180 degree, acquiring first projections with a double angular step size and then the projections in between in reverse order. The mean number of ptychographic steps per projections for the different samples and their standard deviation are provided in Table S2, Supporting Information.

### Ptychographic X‐Ray Computed Tomography—Processing and Tomographic Reconstruction:

In order to recover projection images at each angular position, near‐field ptychographic reconstruction^[^ ^41^ ^]^ were performed using the PtychoShelves package.^[^ ^42^ ^]^ The raw detector frames were cropped to 450 × 450 pixels with the diffraction pattern in the center. A single mode was assumed for the retrieval of the probe. Each projection was reconstructed using an algorithm based on the difference map^[^ ^43^ ^]^ with 1000 iterations, followed by 50 iterations of the linear least‐squares maximum‐likelihood algorithm on compact sets (LSQ‐ML‐c).^[^ ^44^ ^]^ Two independent projections with identical acquisition parameters were used to evaluate the 2D‐resolution with the Fourier ring correlation.^[^ ^45^ ^]^ Employing the 1‐bit criterion,^[^ ^46^ ^]^ a 2D‐resolution between 110 and 125 nm was determined for all samples.

Sample drifts were corrected using the methods detailed in refs. [47] and [48]. For that, a sub‐region within the retrieved projections was selected for the alignment process, which covered the horizontal extent of the specimen. In a second step, constant and linear phase terms were removed in the region‐of‐interest, since they can take arbitrary values in the ptychographic reconstruction and the phase was unwrapped.^[^ ^47^ ^]^ Next, the projections were aligned by maximizing the correlation of their projected phase, which was integrated along the horizontal direction.^[^ ^47^ ^]^ Horizontal shifts were compensated with an iterative sinogram alignment technique based on tomographic consistency.^[^ ^48^ ^]^

Since the parameters used in this study for the horizontal alignment differ from the ones reported in ref. [48], they were briefly summarized. The gradient of the wrapped phase in conjunction with an adapted Ram–Lak filter was employed in the filtered backprojection and the maximum lateral binning‐factor was determined by the sample's horizontal dimension to be 8. The shifts were refined by performing the tomoconsistency alignment iteratively at increasing resolution, achieved by decreasing the binning factor in powers of 2.

After alignment of the projections, phase ramp removal and phase unwrapping were carried out on the whole image.

For every projection, data processing resulted in the complex object transmission function *O*(**r**) for position **r** in the plane perpendicular to the projection direction *z* ^[^ ^25^, ^49^ ^]^

(1) O(r)=T(r)cos(Φ(r))+iT(r)sin(Φ(r))

The absorbing part of the complex transmission function is

(2) $$T\left(r\right)=\exp\left(-\frac{2\pi }{\lambda }\int \beta (r,z)\;dz\right)$$

The phase‐shifting part is

(3) $$\Phi \left(r\right)=\frac{2\pi }{\lambda }\int \delta (r,z)\;dz$$

Hence, two 3D CT data sets were reconstructed with filtered backprojection for both quantities, the refractive index decrement δ as well as the imaginary part of the refractive index β. With the known X‐ray energy (in this case *E* = 6.2 keV, wavelength λ = 2.0Å), the electron density ρ_e_ and absorption coefficient μ distributions in the sample were retrieved from

(4) $$\delta =\frac{2\pi \rho_{e}r_{e}}{k^{2}}$$

and

(5) $$\beta =\frac{\mu }{2k}$$

respectively, with *k* = 2π/ λ.

The reconstructed (geometric) voxel size was 99 nm. The 3D‐resolution was approximately the same as the 2D‐resolution.

### 3D Dictionary Denoising of Ptychographic X‐Ray Computed Tomography Data:

Because noise in the reconstructed attenuation data sets was higher than in the electron density distribution, an image‐based denoising algorithm based on a set of learned image patches, a dictionary, was applied to the reconstructed PXCT data (cf., Figure S6, Supporting Information). The basic idea was that the structural features in the two data sets of the same sample are identical and spatially registered.^[^ ^33^ ^]^ Both applied to the data retrieved in PXCT.

Because the attenuation and electron density distributions originated from the same measurement, the noise in both images was correlated. Since the denoising algorithm was originally designed for uncorrelated noise, a whitening of the data had to be performed. For that, the 2 × 2 covariance matrix Cov, between electron density and attenuation distribution was calculated. With the eigenvectors given in the columns of a matrix *V* and eigenvalues written as diagonal entries of a matrix *L*, the covariance is given by

(6) $$\text{Cov}=VLV^{-1}$$

The linear transformation matrix *M* of Cov is calculated by

(7) $$M=V\sqrt{L}.$$

Multiplying the inverse transformation matrix *M* ^−1^ to the data resulted in uncorrelated values.

In the next step the denoising algorithm was applied. The volumes to be denoised were subdivided into overlapping 3D patches, where the mean value of each patch was subtracted and subsequently the patches were normalized. The size of the image patches was 8 × 8 × 8 pixels with a sliding distance of 1 pixel. The patches from both 3D images were combined to achieve a statistical optimally weighted sum, which was then denoised using a dictionary (learned from noise‐free phantom simulations), where the image patch to be denoised was represented as a linear combination of the dictionary patches. The denoising algorithm terminated if either the small error tolerance, represented by the squared difference between the original image patch and the linearly combined dictionary patches, fell below 0.01, or the number of dictionary patches in the linear combination exceeded 40. After the denoising step, the data set was deconvolved into the two original data sets.^[^ ^33^ ^]^

Back transformation of the uncorrelated data sets into quantitative‐valued data was done by multiplying the transmission matrix *M*, because *M* ^−1^ *M* = 1.

### Image‐Based Material Decomposition:

Given two independent quantities of the sample, basis material images can be retrieved from the microDECT data (measured quantities: μ_low_ and μ_high_)^[^ ^50^ ^]^ and the PXCT data (measured quantities: μ and electron density ρ_e_).^[^ ^26^ ^]^ The aim was to transform the data from the old basis (measured quantities) to a new basis (volume fractions of two desired basis materials, *n*_1_ (I/Br) and *n*_2_ (CPD soft tissue)). The basis vector transformation was done by solving a linear set of equations, which described the coordinates in the old basis (measured quantities) in terms of the new basis (*n*_1_ and *n*_2_). For that, the change‐of‐basis matrices *A* and *A*′ were required, whose columns were the coordinate vectors of the new basis in form of the old basis, which results in

(8) $$\left(\begin{array}{c} \mu_{\text{low}}\\ \mu_{\text{high}} \end{array}\right)=A\cdot \left(\begin{array}{c} n_{1}\\ n_{2} \end{array}\right)=\left(\begin{array}{cc} \mu_{\text{low},1} & \mu_{\text{low},2}\\ \mu_{\text{high},1} & \mu_{\text{high},2} \end{array}\right)\cdot \left(\begin{array}{c} n_{1}\\ n_{2} \end{array}\right)$$

for the microDECT data, and

(9) μρe=A′·n1n2=μ1μ2ρe,1ρe,2·n1n2

in case of the PXCT data. The attenuation coefficient for both low‐ and high‐energy measurement of the CPD soft tissue basis material were taken from the mean values of the total sample volume of an unstained sample. The CPD soft tissue basis material for the PXCT data was as well calculated from the mean electron density and attenuation of the unstained samples. Because the unstained samples differed significantly, especially between the IKI pairs and eosin pairs, the attenuation and electron density values were taken for every sample pair individually from the unstained sample. By retrieving the attenuation coefficient for the CPD soft tissue from measurements, a distinct density for the CPD soft tissue was assumed.

**Table** **1** lists the basis material values of the CPD soft tissue for the different decompositions performed in this work. The attenuation of the CPD soft tissue in the experiments scaled similar with the X‐ray energy as referenced soft tissue (IKI samples: *E* ^−2.75^; eosin samples: *E* ^−3.02^; soft tissue (ICRP):^[^ ^31^ ^]^ *E* ^−2.94^). Because the attenuation coefficients of the IKI and eosin complexes were not known precisely, the respective heavy elements in the X‐ray contrast agents were chosen as the basis material for the stains. The energy‐dependent attenuation coefficient and electron density of the pure elements were well known and where taken from the XMuDat database at the respective mean energies (Figure S5, Supporting Information).^[^ ^31^ ^]^

**Table 1:** Basis material values for the CPD soft tissue for microDECT and PXCT data. The basis materials for the second pairs are provided in brackets if applicable

	MicroDECT		PXCT			
	μ_25kV_ [cm^−1^]	μ_40kV_ [cm^−1^]	μ_6.2keV_ [cm^−1^]		ρ_e_ [*e* ^−^Å^−3^]	
			IKI pair	Eosin pair	IKI pair	Eosin pair
CPD soft tissue	1.56	1.05	18.0 (18.0)	21.9 (23.0)	0.18 (0.19)	0.35 (0.35)

### Statistical Analysis:

Data processing and analysis was performed using Python (including common packages, e.g., numpy^[^ ^51^ ^]^). Besides the dictionary denoising applied to the reconstructed PXCT data, no preprocessing was applied. The presented quantitative results were always given as the mean value of all voxels in a VOI. The size *N* of the VOIs in voxels used to retrieve the presented quantitative results are provided in **Table** **2** for each analysis task. Unless otherwise stated, uncertainties of the quantitative stain concentrations were given as the standard deviation in the background region of the respective data.

**Table 2:** Size of the VOIs *N* in voxels used to retrieve the contrast‐to‐noise ratio for the individual samples in the microDECT measurements as well as the mean volume fractions for both microDECT and PXCT

	MicroDECT			PXCT
	Volume fraction	Contrast‐to‐noise ratio		Volume fraction
		Background region	Sample region	
CWI21	–	8 × 40 × 60	8 × 40 × 60	1 × 175 × 155
CWI22	1 × 90 × 79	20 × 20 × 40	20 × 20 × 40	1 × 175 × 155
CWI31 (cf., Figures 2 and 4)	–	–	–	1 × 220 × 180
CWI32 (cf., Figures 1, 2, and 4)	1 × 264 × 84	20 × 40 × 30	20 × 40 × 30	1 × 175 × 155
CWE21 (cf., Figures 3 and 5)	–	–	–	1 × 200 × 200
CWE22 (cf., Figures 1, 3 and 5)	1 × 60 × 90	20 × 20 × 40	20 × 20 × 40	1 × 190 × 260
CWE31	–	–	–	1 × 148 × 200
CWE32	1 × 100 × 79	10 × 50 × 40	10 × 50 × 40	1 × 120 × 150

**Figure 6:**
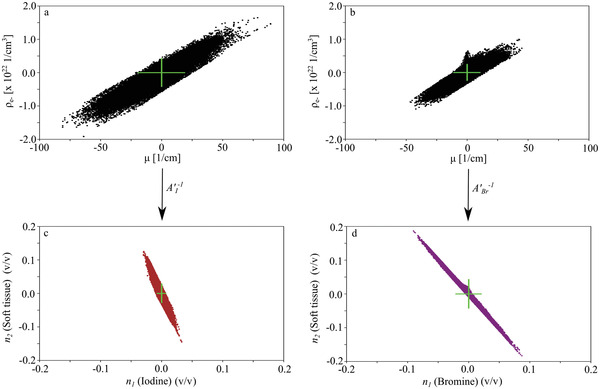
Impact of the different basis vector transformations on the original data. a,b) Measured attenuation and electron density in a background (air) region with PXCT of two samples stained with IKI (a) and eosin (b) (size of VOI: 10 × 30 × 280 voxels for both). Applying the basis vector transformation (via multiplication with $A_{I}^{\prime -1}$ and $A_{\text{Br}}^{\prime -1}$, respectively) induces a stretch and rotation on the original data for both data sets differently, resulting in different standard deviations in the basis material domain for c) the IKI sample and d) the sample stained with eosin.

The standard deviation of the basis material images was on the one hand given by the standard deviation of the original data and additionally depended on the used basis materials. Solving Equations (8) and (9) for *n*_1_ and *n*_2_ was done by inverting the change‐of‐basis matrix. Thus, for example in case of the PXCT data, *A*′^−1^ was multiplied to the measured quantities. **Figure** **6**a,b depict μ and ρ_e_ in a background (vacuum) region (after dictionary denoising) of the IKI stained sample and the sample stained with eosin, respectively. Because both quantities were retrieved from the same measurement, they were correlated. The standard deviations are depicted for both data sets in green. Applying the inverted change‐of‐basis matrix *A*′^−1^, with the respective entries for the iodine and bromine decomposition as given in Table 1 (resulting in different $A_{\text{Br}}^{\prime -1}$ and $A_{I}^{\prime -1}$), stretched and rotated the data differently for both decompositions (Figure 6c,d for the iodine and bromine decomposition, respectively). Thus, the standard deviations in the basis material images of the eosin samples were different compared to the ones of IKI, as illustrated in green in Figure 6c,d. While in case of iodine, the standard deviation especially propagated into the CPD soft‐tissue image, the decomposition into bromine and soft tissue provided an increased standard deviation in both basis material images. The stretch and rotation induced by the image‐based material decomposition were also responsible for the shape of the histograms in Figures 4e,j and 5e,j.

The same applied to the microDECT measurements. However, because the attenuation of bromine and iodine were more similar for the X‐ray energies used for the microDECT measurements, the differences among both decompositions in rotation and stretch were not as high as for the PXCT data, resulting in similar standard deviations for the microDECT basis material images for both X‐ray stains.

## Conflict of Interest

The authors declare no conflict of interest.

## Author Contributions

K.T. and M.B. contributed equally to this work. K.T, M.B., M.D., F.P., and J.H. conceived and designed the research. K.T., M.B., J.B., B.G., M.D., A.D., M.H., and J.H. conducted the experiments. K.T., J.B., B.G., and M.B. analyzed the results. D.M. performed the histological analysis. All authors commented on the manuscript.

## Supporting information

Supporting Information

## Data Availability

The data that support the findings of this study are available from the corresponding author upon reasonable request.

## References

[1] S. J. Schambach , S. Bag , L. Schilling , C. Groden , M. A. Brockmann , Methods 2010, 50, 2. 19706326 10.1016/j.ymeth.2009.08.007

[2] J. Thiesse , E. Namati , J. C. Sieren , A. R. Smith , J. M. Reinhardt E. A. Hoffman , G. McLennan , J. Appl. Physiol. 2010, 109, 1960. 20671036 10.1152/japplphysiol.01322.2009 PMC3006419

[3] P. Bidola , J. Martins de Souza e Silva , K. Achterhold , E. Munkhbaatar , P. J. Jost , A. Meinhardt , K. Taphorn , M‐C. Zdora , F. Pfeiffer , J. Herzen , Sci. Rep. 2019, 9, 1325. 30718557 10.1038/s41598-018-37394-w PMC6362109

[4] M. Müller , I. de Sena Oliveira , S. Allner , S. Ferstl , P. Bidola , K. Mechlem , A. Fehringer , L. Hehn , M. Dierolf , K. Achterhold , B. Gleich , J. U. Hammel , H. Jahn , G. Mayer , F. Pfeiffer , Proc. Natl. Acad. Sci. U. S. A. 2017, 114, 12378. 29109262 10.1073/pnas.1710742114 PMC5703297

[5] B. Metscher , BMC Physiol. 2009, 9, 11. 19545439 10.1186/1472-6793-9-11 PMC2717911

[6] B. Metscher , Dev. Dyn. 2009, 238, 632. 19235724 10.1002/dvdy.21857

[7] A. Shakeri‐Zadeh , A., Expert Opin. Drug Discov. 2019, 14, 849. 31140901 10.1080/17460441.2019.1623203

[8] F. Friedrich , R. G. Beutel , Dev. X‐Ray Tomogr. VI 2008, 7078, 545.

[9] V. Cnudde , B. Masschaele , H. E. V. De Cock , K. Olstad , L. Vlaminck , J. Vlassenbroeck , M. Dierick , Y. D. Witte , L. Van Hoorebeke , P. Jacobs , J. Microsc. 2008, 232, 476. 19094024 10.1111/j.1365-2818.2008.02142.x

[10] J. T. Johnson , M. S. Hansen , I. Wu , L. J. Healy , C. R. Johnson , G. M. Jones , M. R. Capecchi , C. Keller , PLoS Genet. 2006, 2, 471. 10.1371/journal.pgen.0020061 PMC1449902 16683035

[11] S. I. Prajapati , A. Kilcoyne , A. K. Samano , D. P. Green , S. D. McCarthy , B. A. Blackman , M. M. Brady , L. A. Zarzabal , A. K. Tatiparthy , T. J. Sledz , T. Duong , S. Ohshima‐Hosoyama , F. J. Giles , J. E. Michalek , B. P. Rubin , C. Keller , Mol. Imaging Biol. 2011, 13, 493. 20617390 10.1007/s11307-010-0372-3 PMC4770897

[12] M. Busse , M. Müller , M. A. Kimm , S. Ferstl , S. Allner , K. Achterhold , J. Herzen , F. Pfeiffer , Proc. Natl. Acad. Sci. U. S. A. 2018, 115, 2293. 29463748 10.1073/pnas.1720862115 PMC5877988

[13] M. Senter‐Zapata , K. Patel , P. A. Bautista , M. Griffin , J. Michaelson , Y. Yagi , Pathobiology 2016, 83, 140. 27100885 10.1159/000442387

[14] K. Degenhardt , A. C. Wright , D. Horng , A. Padmanabhan , J. A. Epstein , Circ. Cardiovasc. Imaging 2010, 3, 314. 20190279 10.1161/CIRCIMAGING.109.918482 PMC3059892

[15] S. De Bournonville , S. Vangrunderbeeck , G. Kerckhofs , Contrast Media Mol. Imaging 2019, 2019, 8617406. 30944550 10.1155/2019/8617406 PMC6421764

[16] T. Veuthey , G. Herrera , V. I. Dodero , Front. Biosci. (Landmark Ed.) 2014, 19, 91. 24389174 10.2741/4197

[17] R. W.,Biotech. Histochem. 2005, 80, 49.16195171

[18] R. W. Horobin , Biotech. Histochem. 2002, 77, 3. 11991329

[19] M. Müller , M. A. Kimm , S. Ferstl , S. Allner , K. Achterhold , J. Herzen , F. Pfeiffer , M. Busse , Sci. Rep. 2018, 8, 1325. 30552357 10.1038/s41598-018-36067-y PMC6294809

[20] V. M. Pai , M. Kozlowski , D. Donahue , E. Miller , X. Xiao , M. Y. Chen , Z. Yu , P. Connelly , K. Jeffries , H. Wen , J. Anat. 2012, 220, 514. 22360411 10.1111/j.1469-7580.2012.01483.x PMC3323699

[21] C. Dullin , R. Ufartes , E. Larsson , S. Martin , M. Lazzarini , G. Tromba , J. Missbach‐Guentner , D. Pinkert‐Leetsch , D. M. Katschinski , F. Alves Frauke , PLoS One 2017, e0170597. 10.1371/journal.pone.0170597 PMC5298245 28178293

[22] K. M. Lesciotto , S. M. Motch Perrine , M. Kawasaki , T. Stecko , T. M. Ryan , K. Kawasaki , J. T. Richtsmeier , Dev. Dyn. 2020, 249, 573. 31736206 10.1002/dvdy.136 PMC7125040

[23] J. Martins de Souza e Silva , J. Utsch , M. A. Kimm , S. Allner , M. F. Epple , K. Achterhold , F. Pfeiffer , Sci. Rep. 2017, 7, 17387. 29234002 10.1038/s41598-017-17064-z PMC5727238

[24] S. Handschuh , C. J. Beisser , B. Ruthensteiner , B. Metscher , J. Microsc. 2017, 267, 3. 28267884 10.1111/jmi.12543

[25] M. Dierolf , P. Thibault , A. Menzel , C. M. Kewish , K. Jefimovs , I. Schlichting , K. von König , O. Bunk , F. Pfeiffer , Nature 2010, 467, 436. 20864997 10.1038/nature09419

[26] E. Braig , J. Böhm , M. Dierolf , C. Jud , B. Günther , K. Mechlem , S. Allner , T. Sellerer , K. Achterhold , B. Gleich , P. B. Noël , D. Pfeiffer , E. Rummeny , J. Herzen , F. Pfeiffer , Sci. Rep. 2018, 8, 16394. 30401876 10.1038/s41598-018-34809-6 PMC6219573

[27] K. Giewekemeyer , P. Thibault , S. Kalbfleisch , A. Beerlink , C. M. Kewish , M. Dierolf , F. Pfeiffer , T. Salditta , Proc. Natl. Acad. Sci. U. S. A. 2010, 107, 529. 20018650 10.1073/pnas.0905846107 PMC2795774

[28] M. W. M. Jones , K. Elgass , M. D. Junker , M. B. Luu , M. T. Ryan , A. G. Peele , G. A. Van Riessen , Sci. Rep. 2014, 4, 6796. 25348877 10.1038/srep06796 PMC4210942

[29] S. P. Spragg , Electrophoresis in Encyclopedia of Physical Science and Technology (Eds: R. A. Meyers ), Academic Press, San Diego, CA 2003, pp. 363–378.

[30] M. Holler , J. Raabe , R. Wepf , S. H. Shahmoradian , A. Diaz , B. Sarafimov , T. Lachat , H. Walther , M. Vitins , Rev. Sci. Instrum. 2017, 88, 113701. 29195351 10.1063/1.4996092

[31] Nowotny, R. XMuDat: photon attenuation data on PC. https://www‐nds.iaea.org/publications/iaea‐nds/iaea‐nds‐0195.htm (accessed: April 2022).

[32] M.‐C. Zdora , P. Thibault , W. Kuo , V. Fernandez , H. Deyhle , J. Vila‐Comamala , M. P. Olbinado , A. Rack , P. M. Lackie , O. L. Katsamenis , M. J. Lawson , V. Kurtcuoglu , C. Rau , F. Pfeiffer , I. Zanette , Optica 2020, 7, 1221.

[33] K. Mechlem , S. Allner , K. Mei , F. Pfeiffer , P. B. Noël , Med. Imaging 2016: Phys. Med. Imaging 2016, 9783.

[34] M. Holler , J. Ihli , E. H. R. Tsai , F. Nudelman , M. Verezhak , W. D. J. van de Berg , S. H. Shahmoradian , J. Synchrotron Radiat. 2020, 27, 472. 32153287 10.1107/S1600577519017028 PMC7064112

[35] M. Holler , J. Raabe , A. Diaz , M. Guizar‐Sicairos , R. Wepf , M. Odstrcil , F. R. Shaik , V. Panneels , A. Menzel , B. Sarafimov , S. Maag , X. Wang , V. Thominet , H. Walther , T. Lachat , M. Vitins , O. Bunk , Rev. Sci. Instrum. 2018, 89, 4706. 10.1063/1.5020247 29716370

[36] S. H. Shahmoradian , E. H. R. Tsai , A. Diaz , M. Guizar‐Sicairos , J. Raabe , L. Spycher , M. Britschgi , A. Ruf , H. Stahlberg , M. Holler , Sci. Rep. 2017, 7, 6291. 28740127 10.1038/s41598-017-05587-4 PMC5524705

[37] F. Pfeiffer , Nat. Photonics 2018, 12, 9.

[38] I. Johnson , A. Bergamaschi , H. Billich , S. Cartier , R. Dinapoli , D. Greiffenberg , M. Guizar‐Sicairos , B. Henrich , J. Jungmann , D. Mezza , A. Mozzanica , B. Schmitt , X. Shi , G. Tinti , J. Instrum. 2014, 9, C05032.

[39] G. Tinti , A. Bergamaschi , S. Cartier , R. Dinapoli , D. Greiffenberg , I. Johnson , J. H. Jungmann‐Smith , D. Mezza , A. Mozzanica , B. Schmitt , X. Shi , J. Instrum. 2015, 10, C03011. 10.1063/1.4938166 26724009

[40] P. Thibault , M. Dierolf , O. Bunk , A. Menzel , F. Pfeiffer , Ultramicroscopy 2009, 109, 338. 19201540 10.1016/j.ultramic.2008.12.011

[41] M. Stockmar , P. Cloetens , I. Zanette , B. Enders , M. Dierolf , F. Pfeiffer , P. Thibault , Sci. Rep. 2013, 3, 1927. 23722622 10.1038/srep01927 PMC3668322

[42] K. Wakonig , H.‐C. Stadler , M. Odstrčil , E. H. R. Tsai , A. Diaz , M. Holler , I. Usov , J. Raabe , A. Menzel , M. Guizar‐Si solutioncairos , J. Appl. Crystallogr. 2020, 53, 574. 32280327 10.1107/S1600576720001776 PMC7133065

[43] P. Thibault , M. Dierolf , A. Menzel , O. Bunk , C. David , F. Pfeiffer , Science 2008, 321, 379. 18635796 10.1126/science.1158573

[44] M. Odstrčil , A. Menzel , M. Guizar‐Sicairos , Opt. Express 2018, 26, 3108. 29401843 10.1364/OE.26.003108

[45] W. O. Saxton , W. Baumeister , J. Microsc. 1982, 127, 127. 7120365 10.1111/j.1365-2818.1982.tb00405.x

[46] M. van Heel , M. Schatz , J. Struct. Biol. 2005, 151, 250. 16125414 10.1016/j.jsb.2005.05.009

[47] M. Guizar‐Sicairos , A. Diaz , M. Holler , M. S. Lucas , A. Menzel , R. A. Wepf , O. Bunk , Opt. Express 2011, 19, 21345. 22108985 10.1364/OE.19.021345

[48] M. Guizar‐Sicairos , J. J. Boon , K. Mader , A. Diaz , A. Menzel , O. Bunk , Optica 2015, 2, 259.

[49] D. Attwood , X‐Rays and Extreme Ultraviolet Radiation: Principles and Applications, Cambridge University Press, Cambridge 2017.

[50] R. E. Alvarez , A. Macovski , Phys. Med. Biol. 1976, 21, 733. 967922 10.1088/0031-9155/21/5/002

[51] C. R. Harris , K. J. Millman , S. J. van der Walt , R. Gommers , P. Virtanen , D.Cournapeau, E. Wieser , J. Taylor , S. Berg , N. J. Smith , R. Kern , M. Picus , S. Hoyer , M. H. van Kerkwijk , M. Brett , A. Haldane , J. Fernández del Río , M. Wiebe , P. Peterson , P. Gérard‐Marchant , K. Sheppard , T. Reddy , W. Weckesser , H. Abbasi , C. Gohlke , T. E. Oliphant , Nature 2020, 585, 357. 32939066 10.1038/s41586-020-2649-2 PMC7759461

