# Supplementary material for: X‐ray Stain Localization with Near‐Field Ptychographic Computed Tomography
Source: Adv Sci (Weinh). 2022 Jun 24;9(24):2201723. doi: 10.1002/advs.202201723 (PMC9404393; doi:10.1002/advs.202201723)
Supplement: Supplementary file 1 — Supporting Information [file ADVS-9-2201723-s001.pdf]

ADVANCED SCIENCE
Open Access

Supporting Information

for *Adv. Sci.*, DOI 10.1002/advs.202201723

X-ray Stain Localization with Near-Field Ptychographic Computed Tomography

Kirsten Taphorn, Madleen Busse, Johannes Brantl, Benedikt Günther, Ana Diaz, Mirko Holler, Martin Dierolf, Doris Mayr, Franz Pfeiffer and Julia Herzen*

Supporting Information

X-ray Stain Localization with Near-Field Ptychographic Computed Tomography

Kirsten Taphorn[,1], Madleen Busse[1], Johannes Brantl, Benedikt Günther, Ana Diaz, Mirko Holler, Martin Dierolf, Doris Mayr, Franz Pfeiffer, and Julia Herzen*

K. Taphorn, Dr. M. Busse, J. Brantl, Dr. B. Günther, Dr. M. Dierolf, Prof. Dr. F. Pfeiffer and Prof. Dr. J. Herzen
Chair of Biomedical Physics, Department of Physics, School of Natural Sciences, Technical University of Munich, 85748 Garching, Germany
and
Munich Institute of Biomedical Engineering (MIBE), Technical University of Munich, 85748 Garching, Germany
Email: kirsten.taphorn@tum.de

Dr. A. Diaz and Dr. M. Holler
Paul Scherrer Institute, 5232 Villigen PSI, Switzerland

Prof. Dr. D. Mayr
Institute of Pathology, Ludwig-Maximilians-University, 80337 Munich, Germany

Prof. Dr. F. Pfeiffer
Department of Diagnostic and Interventional Radiology, School of Medicine & Klinikum rechts der Isar, Technical University of Munich, 81675 Munich, Germany
and
Institute for Advanced Study, Technical University of Munich, 85748 Garching, Germany

1. These authors contributed equally to this work.

$$2\,KI + I_2 \rightleftharpoons KI_3 + KI \rightleftharpoons K_2I_4$$

Scheme S1: Chemical equilibria of an aqueous iodine potassium iodide (IKI) solution.

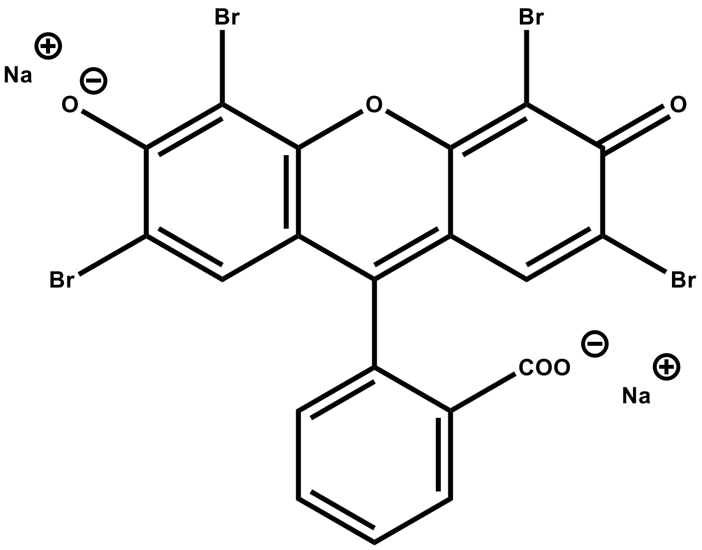

Scheme S2: Chemical structure of Eosin Y (Eosin) disodium salt.

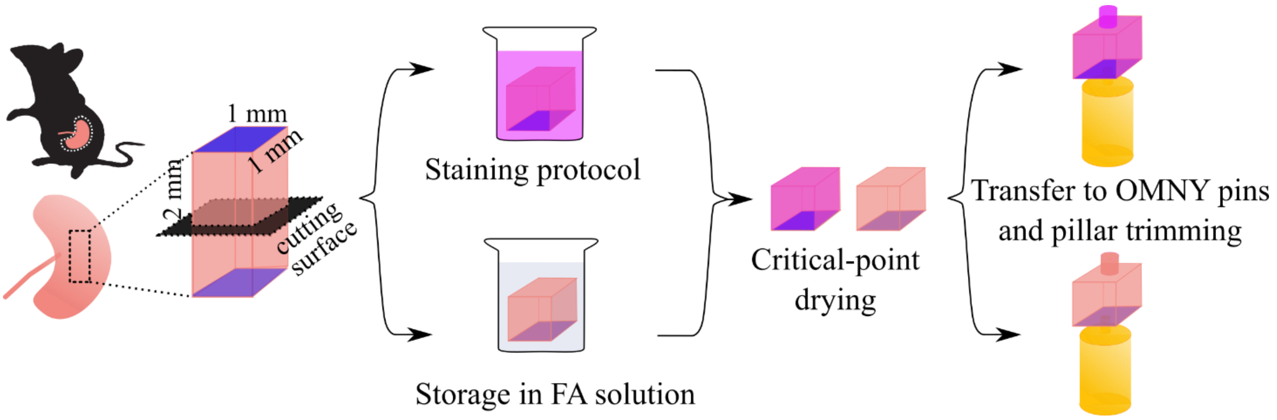

Figure S1: Illustration of the sample preparation. Cubes were cut from the resected mouse kidneys. While one cube underwent the staining protocol, the other one was stored in formaldehyde solution. Afterwards, both cubes were transferred to critical-point-drying and subsequently mounted onto OMNY pins. A pillar on the top was trimmed (diameters for the individual samples provided in Table S1, Supporting Information)

	CWI21 (unstained)	CWI22	CWI32	CWE22	CWE32
**Contrast-to-noise ratio**	32.03	111.99	98.63	64.67	58.44

Table S1: Contrast-to-noise ratios (CNR) for the high energy microDECT measurement for the sample stained with IKI (CWI22, CWI32) and Eosin (CWE22, CWE32) and an unstained sample (CWI21). Sample names marked in green are used for the illustrations in the main article.

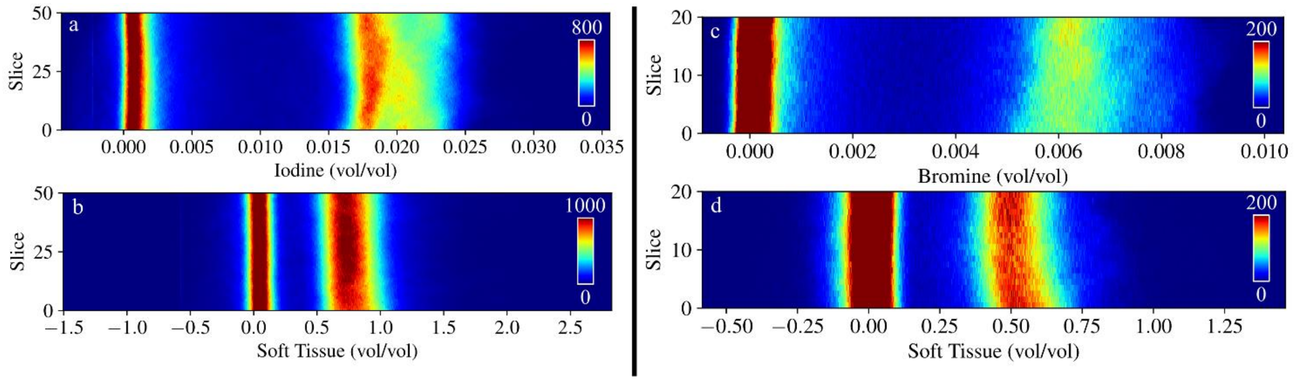

Figure S2: Slice-wise histogram of the three-dimensional basis material images (iodine/bromine (a/c) and CPD soft tissue (b, d)) obtained with dual-energy micro computed tomography (microDECT) of a murine kidney piece stained with IKI (a, b) and Eosin Y (c, d).

**Sample ID**	**Stain**	**Diameter [µm]**	**Horizontal FOV [µm]**	**Vertical FOV [µm]**	**Number of proj.**	**Number of ptycho. Steps**
**CWI21**	No stain	60	66	35	767	143±2
**CWI22**	IKI	65	82	30	930	150±5
**CWI31**	No stain	70	86	40	1027	216±1
**CWI32**	IKI	80	103	32	1160	206±1
**CWE21**	No stain	60	85	45	1068	239±2
**CWE22**	Eosin	60	80	43	1068	215±4
**CWE31**	No stain	60	73	30	930	137±2
**CWE32**	Eosin	60	78	60	930	293±2

Table S2: Overview of the ptychographic X-ray computed tomography (PXCT) parameters for the individual samples. Sample names marked in green are used for the illustrations in the main article.

Conventional Histology

Conventional histology was performed on a fresh mouse kidney, which was fixated in 2% formalin for 8 hours and then embedded in paraffin wax. Subsequently, it was cut along the sagittal (long) axis into slices with a thickness of 4 µm. Afterwards the slides were stained using standard Haematoxylin (Waldeck) and Eosin (Sigma Aldrich). Microscopic images were taken with the LEICA DM 2500 (Leica, Germany) (Fig. 3 e: 12.5x magnification; Fig. 3 f: 200x magnification).

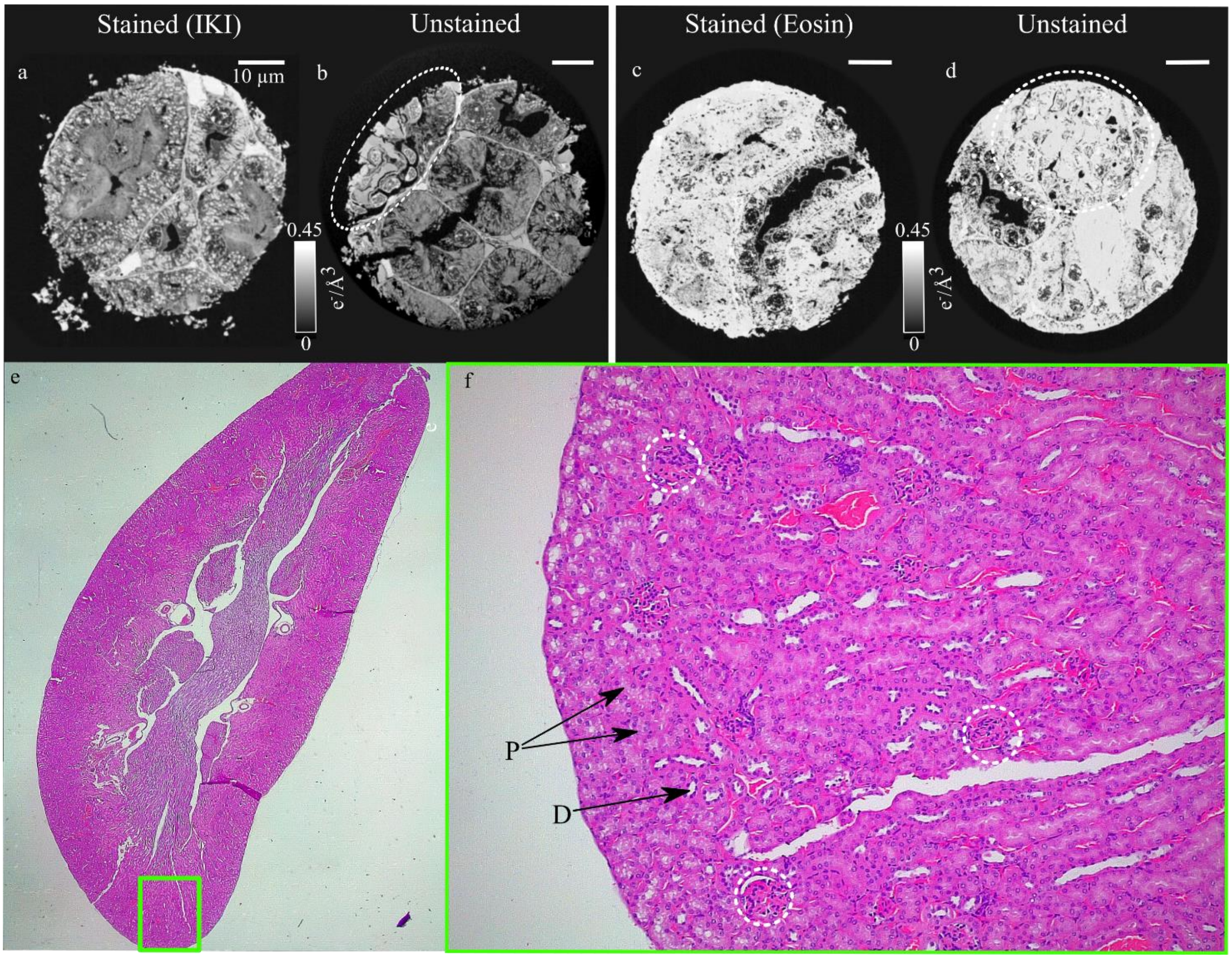

Figure S3: Electron density slices of the stained and unstained samples of the second pairs (IKI (a, b) and Eosin (c, d), respectively). High visual similarity to the sample pairs presented in the main article is given (cf. Fig. 2 and Fig. 3 in the main article). All scalebars 10 µm. e, f: Histological slice of a mouse kidney stained with Eosin and Haematoxylin (location of zoom-in f in kidney indicated in green in e). Annotation: Dotted circles: glomerulus; P: proximal tubules; D: distal tubules

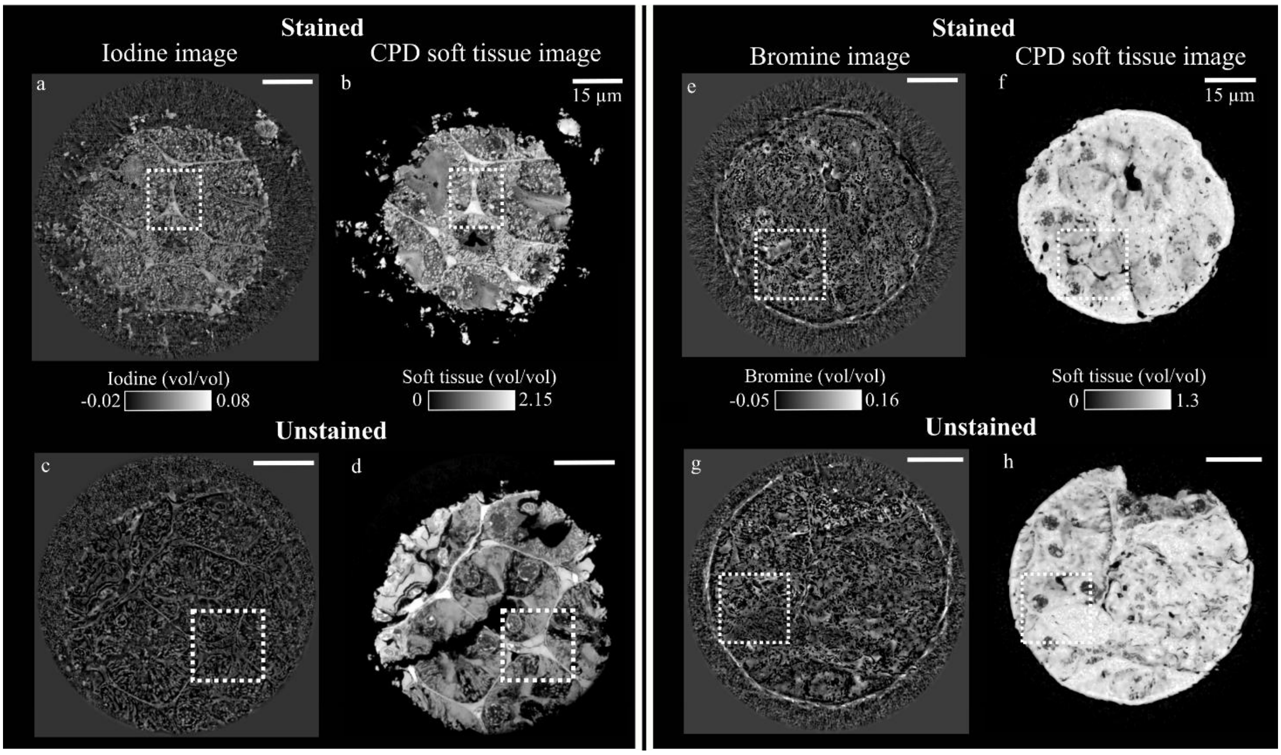

Figure S4: Material decomposition of the second samples pairs for IKI (a-d) and Eosin (e-h), respectively. a, b: Decomposition of the sample stained with IKI. c, d: Decomposition of the unstained sample reference of the second IKI sample pair. e, f: Decomposition of the sample stained with Eosin. g, h: Decomposition of the unstained sample of the second Eosin pair. The dotted rectangles indicate the volume-of-interest for the determination of the basis materials' mean volume fractions as provided in the main article.

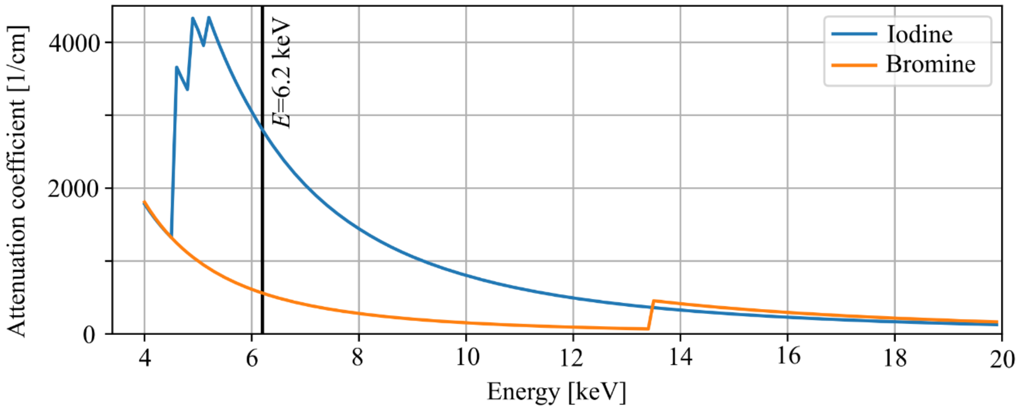

Figure S5: Attenuation coefficients of iodine (blue) and bromine (orange). The beam energy for the PXCT measurements was 6.2 keV. The mean energies of the microDECT scans were 14.7 keV and 18.3 keV, for low and high energy measurement, respectively. Data from [1].

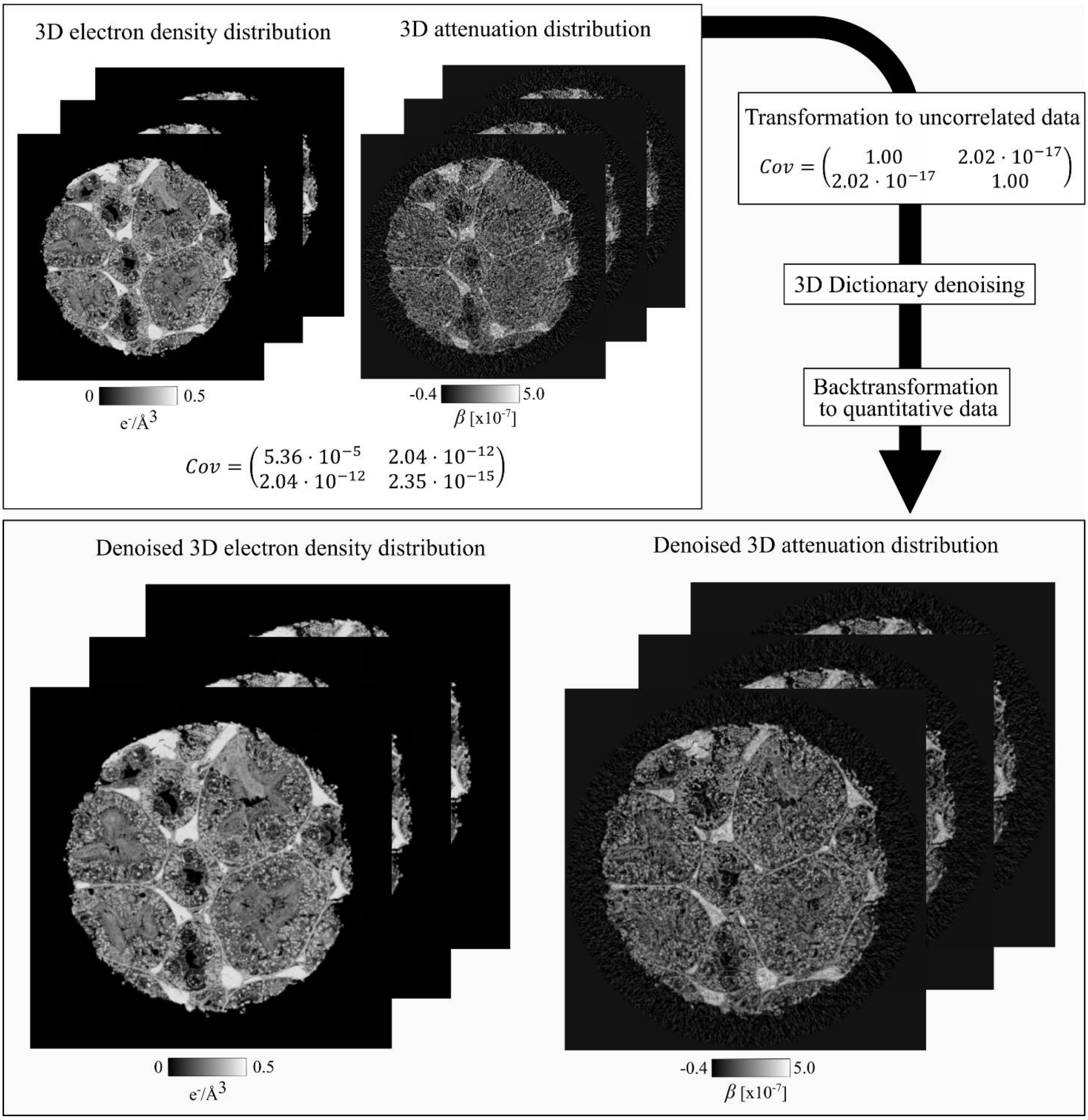

Figure S6: Workflow from the original data (top left) to denoised quantitative PXCT data (on the example of a sample stained with IKI (Table S1, Supporting Information: CWI32)). The 3D electron density and attenuation distributions are correlated. Via a transformation based on their covariance matrix, the data was uncorrelated and subsequently a 3D dictionary denoising algorithm was applied.[2] By a back transformation to quantitative data, denoised electron density and attenuation distributions were achieved (bottom).

References

[1] Nowotny, R. XMuDat: photon attenuation data on PC. International Atomic Agency, Vienna, Austria [Online]. Available: https://www-nds.iaea.org/publications/iaea-nds/iaea-nds-0195.htm.

[2] K. Mechlem, S. Allner, K. Mei, F. Pfeiffer, P. B. Noël *Med. Imaging 2016: Phys. Med. Imaging* **2016**, 9783.
